# Genetic variation in the first intron and exon of the *myostatin* gene in several Indonesian cattle populations

**DOI:** 10.14202/vetworld.2021.1197-1201

**Published:** 2021-05-19

**Authors:** Peni Wahyu Prihandini, Almira Primasari, Aryogi Aryogi, Jauhari Efendy, Muchamad Luthfi, Dicky Pamungkas, Dwi Nur Happy Hariyono

**Affiliations:** 1 Beef Cattle Research Institute of Grati, Pasuruan, Indonesia; 2 Department of Animal Science, Faculty of Agriculture, Universitas Khairun, Ternate, Indonesia

**Keywords:** beef cattle, double muscling, *myostatin* gene, polymorphisms, single-nucleotide polymorphism

## Abstract

**Background and Aim:**

*Myostatin* (*MSTN*), a member of the transforming growth factor-b family, is a negative regulator of muscle mass. This study aimed to detect the genetic variation of the 1160 bp fragment of exon 1 and part of intron 1 of the *MSTN* gene in several cattle populations raised in Indonesia.

**Materials and Methods:**

Polymerase chain reaction products of the *MSTN* gene amplified from 92 animals representing 10 cattle populations (Peranakan Ongole [PO], Belgian Blue x PO cross, Rambon, PO x Bali cross, Jabres, Galekan, Sragen, Donggala, Madura, and Bali) were sequenced, compared, and aligned with bovine *MSTN* of *Bos taurus* (GenBank Acc. No. AF320998.1) and *Bos indicus* (GenBank Acc. No. AY794986.1).

**Results:**

Four nucleotide substitutions (nt 1045 and 1066 in intron 1; nt 262 and 418 in exon 1) and two indels (nt 807 and 869 in intron 1) were synonymous mutations. Among these substitutions, only the nt 262G>C and nt 418A>G loci were polymorphic in all populations, except Bali cattle. The frequencies of the nt 262C (0.82) and nt 418A (0.65) alleles were highest. For the nt 262G>C locus, the CC genotype had the highest frequency (0.66) followed by GC (0.30) and CC (0.03). For the nt 418A>G locus, the AG genotype had the highest frequency (0.52) followed by AA (0.39) and GG (0.09).

**Conclusion:**

The results, showing genetic variations in exon 1 and intron 1 of the *MSTN* gene, might be helpful for future association studies.

## Introduction

Genetic improvement for economically important traits in livestock depends primarily on selective breeding using superior phenotypes. Current technologies using marker-assisted selection (MAS) enable scientists to improve the accuracy and efficiency of traditional selection methods and enhance the understanding of DNA polymorphisms that affect animal production traits [1]. If a single-nucleotide polymorphism (SNP) affects particular traits of interest, it could be used for MAS in livestock breeding programs [2]. Therefore, knowledge of genetic polymorphisms that are significantly associated with production traits of farm animals is of great interest to animal breeders.

In this study, *myostatin* (*MSTN*) was examined as a possible genetic marker in cattle breeding. The *MSTN* gene is a member of the transforming growth factor-b family, which acts as a negative regulator of skeletal muscle mass deposition and plays an important role in mammalian postnatal muscle development [3,4]. The bovine *MSTN* gene is located at 3.1 cM (centimorgan) of the centromeric region on chromosome 2 (BTA2), next to TGLA44 microsatellites [5-7]. *MSTN*-deficient animals exhibit an increase in skeletal muscle mass with both hyperplasia and hypertrophy. This increased muscle mass results from the influence of *MSTN* on cell cycle control genes that cause myogenic progenitor cells to withdraw from the cell cycle permanently [8]. Furthermore, *MSTN* is an inhibitor of progenitor proliferation of skeletal muscle cells during animal development until the formation of skeletal muscle size after birth [9]. Mutations in the *MSTN* gene can affect the proper regulation of skeletal muscle mass, which is important for improved meat production traits. Many previous studies have investigated genetic variations of the *MSTN* gene not only in cattle [3,6,10-14], but also in other livestock species, including goats [15], geese [16], chickens [17], and sheep [18]. Several cattle breeds are characterized by a double-muscling (DBM) phenotype, which increases muscle mass due to loss-of-function mutations in bovine *MSTN* [3,10,19,20]. In Belgian Blue cattle, a DBM phenotype is characterized by an 11 bp deletion (c.821_831del11) in the third exon [3,6], which has also been found in Asturiana de Los Valles, Blonde d’Aquitaine, Limousine, Parthenaise, and Rubia Gallega cattle breeds [6,10]. In Piedmontese cattle *MTSN*, a missense mutation in exon 3 (c.938 G>A) changes a cysteine residue to a tyrosine, causing a DBM phenotype [10]. The same mutation has also been identified in Gascon cattle [10,12].

Up to now, few studies have investigated the *MSTN* variations in Indonesian cattle. Khasanah *et al*. [13] only investigated the promoter region of bovine *MSTN* in Bali cattle, while Anwar *et al*. [21] only investigated the F94L loci of bovine *MSTN*. To date, there is no literature available on the polymorphisms of the *MSTN* gene in several Indonesian local cattle types, such as Donggala, Galekan, Sragen, and Madura, analyzed in the present study. Furthermore, because of its important functions, *MSTN* is considered a candidate gene for growth-related traits in meat-producing animal species [13,16,22].

Therefore, this study investigated the genetic polymorphisms of the *MSTN* gene in several cattle populations raised in Indonesia.

## Materials and Methods

### Ethical approval

The experimental procedures were carried out following the guidelines established by the Ministry of Agriculture of Indonesia. The Indonesian Agency for Agricultural Research and Development approved the procedures (Balitbangtan/Lolitsapi/Rm/09/2020).

### Study period and location

The study was conducted from April to September 2019. The Peranakan Ongole (PO), Belgian Blue × PO cross (BP), PO × Bali cross (PB), and Bali (BL) cattle were obtained from the Beef Cattle Research Institute of Grati, East Java, while the Rambon (RM), Jabres (JB), Galekan (GK), Sragen (SR), Donggala (DG), and Madura (MD) cattle were collected from Banyuwangi of East Java Province, Brebes of Central Java Province, Trenggalek of East Java Province, Sragen of Central Java Province, Donggala of Central Sulawesi Province, and Pamekasan of East Java Province, respectively. Samples were processed at the Indonesian Beef Cattle Research Station.

### Animals and sample collection

This study involved 92 animals from various cattle populations farmed in Indonesia. Breeds evaluated included PO (n=10), BP (n=8), RM (n=10), PB (n=10), JB (n=10), GK (n=10), SR (n=10), DG (n=9), MD (n=10), and BL (n=5). All the studied breeds, except BP and PB crosses, have been identified as local Indonesian cattle by the Ministry of Agriculture of the Republic of Indonesia [23] and are included in the national genetic resources program due to their economic importance. Blood samples, taken from the jugular vein, were collected in 3 mL tubes containing EDTA as an anticoagulant and kept at 4°C until analyzed.

### DNA preparation and polymerase chain reaction (PCR) amplification

Genomic DNA was extracted from the blood samples using a gSYNC™ DNA extraction kit (Geneaid, New Taipei City, Taiwan) and stored at −20°C before analysis. A fragment of 1160 bp covering part of intron 1 and exon 1 of bovine *MSTN* was amplified using PCR. The primer sequences selected were MSTN_F, 5’-AGTATAAAAGATTCACTGGTGTGGC-3’ and MSTN_R, 5’-TGTGTTTACTTCCTTATTGCTCTTACTA-3’ [11] based on the bovine *MSTN* sequence (GenBank Acc. No. AF320998). The PCR reaction was performed using SensoQuest (Germany) and made up of 2 μL of template DNA (10-100 ng), 0.5 μL of each primer (0.25 μM), 12.5 μL PCR kit diluent (2x My Taq HS Red Mix gSYNCTMPCR Kit-Bioline-London), and 9.5 μL ddH_2_O for a total volume of 25 μL. The thermal cycling included an initial denaturation at 95°C for 5 min, followed by 35 cycles of 94°C for 45 s, 57°C for 45 s, and 72°C for 60 s, with a final extension step at 72°C for 5 min. The PCR products were sequenced using an ABI 3730xl genetic analyzer (Applied Biosystems, Foster City, CA, USA).

### Statistical analysis

The *MSTN* gene sequencing results were manually checked and edited using BioEdit software [24] and aligned to the reference bovine *MSTN* (*Bos taurus*, GenBank Acc. No. AF320998.1 and *Bos indicus*, GenBank Acc. No. AY794986.1) using the ClustalW program [25]. Allelic and genotypic frequencies and Chi-square test were performed using POPGENE 1.32 software (https://sites.ualberta.ca/~fyeh/index.html) [26].

## Results

**Figure-1:**
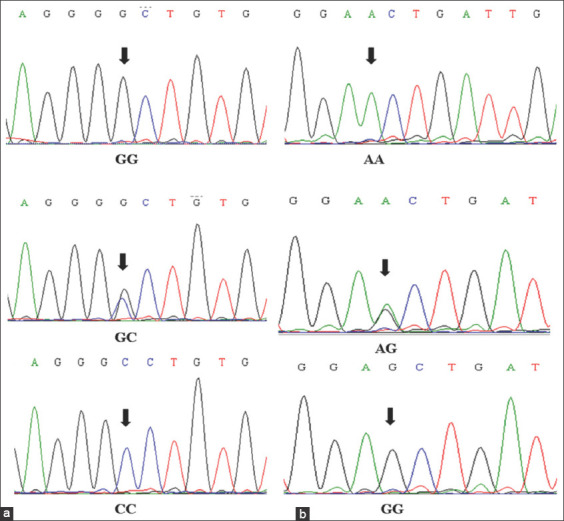
Chromatogram of the *MSTN* gene exon 1. (a) Variation at nt 216 (G>C transition), from top to bottom: Homozygote GG, heterozygote GC, and homozygote CC. (b) Variation at nt 418 (A>G transition), from top to bottom: Homozygote AA, heterozygote AG, and homozygote GG.

The 1160 bp fragment, including part of intron 1 and exon 1, of the bovine *MSTN* gene, was successfully sequenced for 92 individual animals from 10 cattle populations in Indonesia. *B. indicus* cattle breeds are prevalent in Indonesia, but several breeds come from a mixture of *B. indicus* and *B. taurus*. For that reason, the resulted sequences were compared to zebrine and bovine *MSTN* sequences deposited to GenBank (*B. indicus* Acc. No. AY794986 and *B*. *taurus* Acc. No. AF320998). By alignment of those sequences, we observed four nucleotide substitutions (nt 1045 and 1066 in intron 1; nt 262 and 418 in exon 1) and two indels (nt 807 and 869 in intron 1), which were synonymous mutations (Table-1). However, only nt 262 and 418 were polymorphic across the Indonesian cattle populations (Figure-1). These two SNPs generated three genotypes each, which were homozygote GG, heterozygote GC, and homozygote CC for the SNP (G>C) at nt 262, and homozygote AA, heterozygote AG, and homozygote GG for the SNP (A>G) at nt 418.

**Table-1:** Polymorphisms of *myostatin* gene detected in this study.

Base position (AF320998.1)	Location	*Bos taurus* sequence (AF320998.1)	*Bos indicus* sequence (AY794986.1)	Indonesian cattle
262	Exon 1	G	G	G/C
418		A	G	A/G
807	Intron 1	T	-	-
869		Insertion	T	-
1045		T	-	T
1066		G	T	G

Table-2 shows the allelic and genotypic frequencies and Chi-square test results of the *MSTN* loci in the 10 cattle populations (nt 262G>C locus) and Table-3 (nt 418A>G locus). In the overall populations, the ­frequencies of nt 262C (0.82) and nt 418A (0.65) alleles were the highest. For the nt 262G>C locus, the CC genotype had the highest frequency (0.66) followed by GC (0.30) and CC (0.03) genotypes. For the nt 418A>G locus, AG genotypes had the highest frequency (0.52) followed by AA (0.39) and GG (0.09) genotypes. Interestingly, there was no variation in Bali cattle for both loci. All the tested animals belonged to the GG genotype for the nt 262G>C locus and the AA genotype for the nt 418A>G locus. The Chi-square test showed that all the studied populations, except BP and BL, were found to be in Hardy–Weinberg equilibrium for both loci (p>0.05).

**Table-2:** Allelic and genotypic frequencies and Chi-square test of the *MSTN* nt 262G>C in the studied populations.

Population	Genotype			Allele		χ^2^ (HWE)	p-value
	
	GG	GC	CC	G	C		
PO	0.50	0.30	0.20	0.65	0.35	1.5879	0.208
BP	0.88	0.12	0.00	0.94	0.06	0.0000	0.000
RM	0.50	0.50	0.00	0.75	0.25	0.8571	0.355
PB	0.60	0.40	0.00	0.80	0.20	0.4500	0.502
JB	0.80	0.20	0.00	0.90	0.10	0.0588	0.808
GK	0.60	0.40	0.00	0.80	0.20	0.4500	0.502
SR	0.80	0.20	0.00	0.90	0.10	0.0588	0.808
DG	0.33	0.56	0.11	0.61	0.39	0.1108	0.739
MD	0.80	0.20	0.00	0.90	0.10	0.0588	0.808
BL	1.00	0.00	0.00	1.00	0.00	-	-
All	0.66	0.30	0.03	0.82	0.18	0.0021	0.963

HWE=Hardy–Weinberg equilibrium, PO=Peranakan Ongole cattle, BP=Belgian Blue X PO cross cattle, RM=Rambon cattle, PB=PO X Bali cross cattle, JB=Jabres cattle, GK=Galekan cattle, SR=Sragen cattle, DG=Donggala cattle, MD=Madura cattle, BL=Bali cattle.

**Table-3:** Allelic and genotypic frequencies and Chi-square test of the *MSTN* nt 418A>G in the studied populations.

Population	Genotype			Allele		χ^2^ (HWE)	p-value
	
	AA	AG	GG	A	G		
PO	0.30	0.50	0.20	0.55	0.45	0.0182	0.893
BP	0.50	0.50	0.00	0.75	0.25	0.6364	0.425
RM	0.20	0.70	0.10	0.55	0.45	1.3136	0.252
PB	0.40	0.60	0.00	0.70	0.30	1.4835	0.223
JB	0.20	0.70	0.10	0.55	0.45	1.3136	0.252
GK	0.50	0.40	0.10	0.70	0.30	0.1055	0.745
SR	0.40	0.60	0.00	0.70	0.30	1.4835	0.223
DG	0.33	0.56	0.11	0.61	0.39	0.1108	0.739
MD	0.40	0.40	0.20	0.60	0.40	0.4870	0.485
BL	1.00	0.00	0.00	1.00	0.00	-	-
All	0.39	0.52	0.09	0.65	0.35	1.9263	0.165

HWE=Hardy–Weinberg equilibrium, PO=Peranakan Ongole cattle, BP=Belgian Blue X PO cross cattle, RM=Rambon cattle, PB=PO X Bali cross cattle, JB=Jabres cattle, GK=Galekan cattle, SR=Sragen cattle, DG=Donggala cattle, MD=Madura cattle, BL=Bali cattle.

## Discussion

Genetic improvement of farm animals has gained much attention in recent years, particularly for growth performance and carcass traits which are economically important domestic animal characteristics, due to their contributions to farm profitability. Many studies have explored candidate genes or loci related to these economic traits in many domestic animals over the past decades. For instance, *MSTN* is a vital candidate gene responsible for growth and carcass traits in cattle. Gene mutations that contribute to loss of *MSTN* function have been linked to the occurrence of DBM in cattle [10].

This study showed polymorphisms in part of intron 1 and exon 1 of the *MSTN* gene in Indonesian cattle sequences compared to the cited GenBank sequences (Table-1). All the mutations (nt 262, 418, 807, 869, 1045, and 1066) observed in this study were synonymous and reported previously in Qinchuan and Red Angus cattle breeds [11]. Tantia *et al*. [27] sequenced the *MSTN* gene of Indian *B. indicus* breeds (i.e., Rathi, Deoni, Idduki, and Vatakara) and compared their sequences to *B. taurus* (GenBank Acc. AB076403). Their study found two SNPs in exon 1 of the *MSTN* gene, including nt 244 and nt 400, equal to nt 262 and nt 418 detected in the present study. It is notable that Tantia *et al*. [27] determined base position according to GenBank Acc. No. AB076403 of *B. taurus*, while the present study used GenBank Acc. No. AF320998.1 of *B. taurus*. Dunner *et al*. [12] detected two SNPs (nt 267A>G and nt 324C>T) in exon 1 of the *MSTN* gene in some French breeds (Aubrac, Badaize, and Salers), of which nt 267 (GenBank Acc. No. AF019620.1) is equal to nt 418 identified in this study. A nucleotide substitution at nt 267 has also been reported previously in Nellore cattle [28]. The polymorphisms identified in this study provide data for future association research.

Several studies have pointed out six SNPs in the bovine *MSTN* gene responsible for a DBM phenotype, including nt 419 (del7-ins10) in Maine-Anjou cattle, nt 610 (C→T) in Carolaise and Limousine cattle, nt 676 (G→T) in Maine-Anjou cattle, nt 821 (del11) in Asturiana de Los Valles, Belgian Blue, Limousine, Parthenaise, and Rubia Gallega cattle, nt 874 (G→T) in Marchigiana cattle, and nt 938 (G→A) in Gascon and Piedmontese cattle [3,10,19,20]. Unfortunately, the primers used in this study did not cover these loci. Haruna *et al*. [14] found seven nucleotide variations (c.373+751G>T, c.373+803 T>G, c.373+877A>G, c.373+895G>C, c.374-909C>T, c.374-842G>C, c.374-812A>G) in intron 1 of *MSTN* in New Zealand cattle breeds, of which two variants (c.748-281C>G and c.748-352C>T) have been reported in Qinchuan and Red Angus cattle [11]. In contrast to the results of this study, Haruna *et al*. [14] did not observe any nucleotide variation in exon 1 of the *MSTN* gene in New Zealand cattle.

## Conclusion

Our preliminary study suggests polymorphisms in exon 1 and part of intron 1 of the *MSTN* gene in Indonesia cattle. Further studies are needed to evaluate the effects of these polymorphisms on ­growth-related traits in a larger sample size to create a selection tool for improved growth traits in breeding programs.

## Authors’ Contributions

PWP, AA, JE, ML, and DP designed the study and collected the samples. AP collected samples and performed laboratory analysis. DNHH analyzed the data and wrote the manuscript. All authors have read and approved the final manuscript.
